# Antimicrobial Regimens in Cement Spacers for Periprosthetic Joint Infections: A Critical Review

**DOI:** 10.3390/antibiotics13080772

**Published:** 2024-08-15

**Authors:** Symeon Naoum, Christos Koutserimpas, Ioannis Pantekidis, Vasileios Giovanoulis, Enejd Veizi, Maria Piagkou, Petros Ioannou, George Samonis, Aglaia Domouchtsidou, Andreas G. Tsantes, Dimitrios V. Papadopoulos

**Affiliations:** 1 Department of Trauma and Orthopaedics, Royal Berkshire Hospital, Reading RG1 5AN, UK; symeon.naoum@royalberkshire.nhs.uk; 2 Orthopaedic Surgery and Sports Medicine Department, Croix-Rousse Hospital, University Hospital, 69317 Lyon, France; 3 Department of Anatomy, School of Medicine, Faculty of Health Sciences, National and Kapodistrian University of Athens, 75 Mikras Asias Str., Goudi, 11527 Athens, Greece; mapian@med.uoa.gr; 4 Department of Trauma and Orthopaedics, Guy’s and St. Thomas’ NHS Foundation Trust, London SE1 7EH, UK; 5 Department of Orthopaedic Surgery, Hôpital Henri Mondor, AP-HP, Université Paris Est Créteil (UPEC), 94010 Creteil, France; vgiovanoulis@hopital-dcss.org; 6 Department of Orthopedics and Traumatology, Yıldırım Beyazıt University, Ankara City Hospital, Ankara 2367, Turkey; eveizi@hacettepe.edu.tr; 7 School of Medicine, University of Crete, 71003 Heraklion, Greecesamonis@med.uoc.gr (G.S.); 8 First Department of Medical Oncology, Metropolitan Hospital of Neon Faliron, 18547 Athens, Greece; 9 Microbiology Department, “Saint Savvas” Oncology Hospital, 11522 Athens, Greece; ldomouchtsidou@gmail.com (A.D.); agtsantes@med.uoa.gr (A.G.T.); 10 Laboratory of Hematology and Blood Bank Unit, “Attikon” University Hospital, School of Medicine, National and Kapodistrian University of Athens, 12462 Athens, Greece; 11 2nd Academic Department of Orthopaedics, School of Medicine, National and Kapodistrian University of Athens, 14233 Athens, Greece; papadopo@med.uoa.gr

**Keywords:** cement, knee, hip, arthroplasty infections, periprosthetic joint infections, joint reconstruction

## Abstract

Antibiotic-loaded cement spacers (ALCSs) are essential for treating periprosthetic joint infections (PJIs) by providing mechanical support and local antibiotic delivery. The purpose of this review is to comprehensively examine the various types of spacers utilised in the management of periprosthetic joint infections (PJIs), including both static and articulating variants and to analyse the fundamental principles underlying spacer use, their clinical benefits, the selection and administration of antimicrobial agents, appropriate dosages, and potential adverse effects. Articulating spacers, which allow joint mobility, often yield better outcomes than static ones. Spacer pharmacokinetics are vital for maintaining therapeutic antibiotic levels, influenced by cement porosity, mixing techniques, and the contact area. Antibiotic choice depends on heat stability, solubility, and impact on cement’s mechanical properties. Mechanical properties are crucial, as spacers must withstand physical stresses, with antibiotics potentially affecting these properties. Complications, such as tissue damage and systemic toxicity, are discussed, along with mitigation strategies. Future advancements include surface modifications and novel carriers to enhance biofilm management and infection control.

## 1. Introduction

In the past two decades, joint replacements have increased globally, and this trend is expected to continue [1,2,3]. Consequently, revision arthroplasties are also rising, with periprosthetic joint infections (PJIs) being the primary cause [1,2,3].

PJIs can be classified based on the postoperative onset time as early-onset—usually under 3 months from the operation, delayed-onset—after 3 months and before 12–24 months—and late-onset—if presented more than 12–24 months postoperatively [4]. Early-onset PJI is usually due to intraoperative contamination with virulent microorganisms, delayed-onset occurs intraoperatively by microorganisms with less virulence, and late-onset is most commonly caused by hematogenous spread or intraoperatively by very low virulence microorganisms [1,4,5,6].

In PJIs, microorganisms can strongly adhere to the implants. Once the initial adherence is achieved, biofilms are created. Biofilms are complex microorganism clusters in an extracellular matrix on the implant’s surface that create a protective structure for pathogens. They also weaken antimicrobial and host immune cell penetration [4,7,8,9]; thus, making PJI difficult to treat without surgical intervention. Treatment of PJI requires a combination of surgical and antimicrobial therapy, with long-term antibiotic administration. Different surgical approaches have been developed throughout the years. They can be categorised into four main groups: debridement antibiotics and implant retention (DAIR), one-stage revision arthroplasty, two-stage revision arthroplasty with or without antibiotic-loaded cement spacer (ALCSs), and arthroplasty resection without reimplantation, while suppression antibiotic therapy and amputation are very rarely used anymore. Nevertheless, they can still be performed in difficult-to-treat cases. For delayed-onset and late-onset PJIs, a two-stage revision arthroplasty with the ALCS is considered the standard treatment [4,5]. In two-stage revision arthroplasties, ALCSs are combined with systemic administration of antibiotics to enhance microorganism eradication. There are different types of ALCSs, and their use and effectiveness differ depending on the PJI.

The present narrative review provides insights into the antimicrobial agents used in cement spacers during the management of PJIs. We analyse the basic principles of spacers used and their pharmacokinetics, the antibiotics with their parameters, the properties of the ALCS, and the overall known complications, providing an up-to-date overview of the current literature.

## 2. Basic Principles and Types of Spacers

### 2.1. The Role of Antibiotic-Loaded Cement Spacers in PJIs

The role of the ALCS in the treatment of PJIs is both mechanical and pharmaceutical [5,10,11]. Mechanically, the cement spacer fills the void left after debridement and the removal of the infected prosthesis. This prevents extensive scarring, maintains bone quality and limb length, and facilitates the final implantation of a new prosthesis [10,12,13,14]. Prior to the use of cement spacers in two-stage revision arthroplasty for PJIs, prolonged immobilization between surgeries often led to muscle atrophy, soft tissue contraction, limb shortening, and joint stiffness, making the second stage surgery very challenging [10,13,15,16,17].

The most critical function of the spacer, in managing PJI, is serving as a potent local antibiotic carrier. Systemic antibiotic administration after the first stage of surgery (prosthesis removal) is primarily effective against free-floating microorganisms [9,18]. However, in delayed or late-stage PJI, biofilms are well-established. Treating microorganisms within these biofilms requires high local antibiotic concentrations, which would be toxic if achieved through systemic administration [10,18]. The ALCS is the only method to achieve such high local antibiotic concentrations without increasing levels in the plasma or urine [5,11,12,18]. With ALCS, the antibiotic concentration at the infection site can be up to 700 times higher than with systemic administration [10,19], while avoiding systemic complications [5]. Additionally, the spacer delivers antibiotics directly to tissues with poor blood supply, where systemic administration cannot reach the minimum inhibitory concentration (MIC) needed to eradicate the infection [5].

### 2.2. The Types of Spacers in PJIs

Based on mobility limitations, spacers are classified into articulating and non-articulating (static). Non-articulating spacers can release high local antibiotic concentrations, enhancing patient autonomy and maintaining joint space for subsequent stages [8]. Articulating spacers offer advantages, such as better preservation of bone mass, higher patient satisfaction, prevention of extensor mechanism impairment, and better postoperative patient-reported outcomes [15,20,21]. The basic types of articulating spacers are hand-made, mould-fashioned, commercially-preformed, spacers with additional metal or polyethylene articulating elements, autoclaved prostheses, custom-made and 3D-printed (Table 1). Moreover, they preserve joint mobility between revision stages, reducing muscle atrophy and scar tissue formation within the joint. This leads to improved conditions for the second-stage surgery, resulting in superior functional outcomes and a broader postoperative range of motion [10,11,22]. While systematic reviews and meta-analyses on static and articulating spacers or different types of articulating spacers (Table 1) yield conflicting results, many researchers advocate for the superiority of articulating spacers [11,12,22]. Additionally, the use of static spacers correlates with more extensive surgical approaches during the second revision stage and a higher reoperation rate [22]. Regarding articulating spacers, they are preferred for patients requiring spacers to remain in place for longer than 3 months [23]. Comparisons between various types of articulating spacers indicate their equal effectiveness in treating PJI, providing similar preoperative conditions for the second-stage implantation and comparable functional outcomes for the joint [10,11,24].

## 3. Spacer Pharmacokinetics

The effectiveness of a spacer as a local antibiotic carrier hinges on its capacity to maintain high concentrations of the antibiotic at the infection site for a sufficient duration [5,25]. Local antibiotic levels need to exceed the MIC of the antibiotic against the causative organisms [26,27,28]. These elevated concentrations should be sustained between the two stages of the revision surgery to eradicate the pathogens and prevent infection recurrence or the emergence of resistant strains [26,28,29].

The antibiotic is released from the spacer through diffusion. Initially, the antibiotic elutes from the cement surface, but continued release occurs from the inner cement body via channels, cracks, and cavities formed by friction and compression during weight-bearing [26,30]. The release of antibiotics from polymethylmethacrylate (PMMA) is primarily a surface phenomenon and is independent of the cement’s volume [28]. The diffusion rate is influenced by the dose and type or combination of antibiotics, the cement mixing process, its porosity, and the surface morphology of the spacer that contacts the inflamed tissues [5,25,26].

Studies have shown that the greater the surface area of the cement in contact with surrounding tissues, the higher the rate and amount of antibiotic release from the spacer [5,18,28]. Increased distance between the cement and surrounding tissues results in lower antibiotic concentrations [5]. The type of tissue around the spacer also influences antibiotic elution and diffusion into the inflamed tissues, with cortical bone absorbing less antibiotic than cancellous bone, and cancellous bone absorbing less than hematoma (dead space) between the spacer and bone or soft tissues. Therefore, maximizing contact between the spacer and inflamed tissues is essential [5,18].

In addition, joint motion and tissue absorption capacity can influence the amount of antibiotic released from the spacer. This may explain the lack of correlation between intra-articular and systemic antibiotic concentrations reported in various studies. [26,28,31]. Vacuum mixing improves the mechanical properties of the cement by reducing porosity, thereby decreasing the likelihood of fractures during cyclic loading [8,32]. Hand-mixed cement exhibits increased antibiotic elution; particularly, five times more vancomycin elution and two times more gentamicin. This is attributed to the higher porosity of the PMMA [33]. Vacuum mixing involves blending PMMA powder and liquid components under vacuum pressure to reduce bulk porosity. This process minimises the burst release of antibiotics and enhances the mechanical properties of the solidified cement [34]. Conversely, Neut et al. [35] found that gentamicin release from DePuy CMW and Palamed cement was generally higher when hand-mixed [36]. Increased porosity enhances elution, and partially mixed cements, which have more intact crystals from the mixing process, create a more porous cement with a higher release rate.

However, these cements are unsuitable for prosthesis fixation due to their weak mechanical strength [35]. Dextran has been used as a porosity agent to increase elution rates, with antibiotic release from dextran-containing samples being four times higher than routine samples within the first 48 h, and the duration of elution extending from 6–10 days [37]. The release kinetics of different antibiotics from cement vary widely [8,29]. Vancomycin-impregnated cement spacers have demonstrated significantly better and longer-lasting inhibitory effects on methicillin-resistant *S. aureus* (MRSA) compared to fosfomycin-loaded spacers [38]. The aminoglycoside–glycopeptide combination is designed to prevent bacterial resistance, but a higher proportion of vancomycin is necessary due to its greater release kinetics [31]. In Table 2, the factors affecting antibiotic release from ALCSs are presented.

## 4. Antimicrobial Agents in Spacers

### 4.1. Characteristics of Antibiotics for Use in Cement Spacers

Antibiotics used in spacers must withstand the heat generated during PMMA polymerization (up to 82–83 °C for 12–13 min) to avoid degradation [11,39]. They should be water-soluble to allow diffusion into surrounding tissues post-implantation [11]. The antibiotics must be chemically stable, not reacting with the cement’s components, and should have bactericidal properties even at low concentrations. Additionally, they should have a low MIC for the targeted pathogens, not bind to serum proteins, not cause allergic reactions, and not promote resistant bacteria [18,26].

Powdered antibiotics are preferred over liquid forms, as the latter can hinder polymerization, increase porosity, and reduce the mechanical strength of the spacer [5]. The cement’s compressive strength decreased by 13% with vancomycin, 37% with liquid gentamicin, and 45% when both were used [29]. The molecular size of the antibiotic is crucial, as smaller molecules are more water-soluble, leading to a quicker reduction in inhibition zones [40]. The antimicrobial agent should be added to the cement once it has reached its liquid form: the PMMA monomer and powder are initially mixed together and once the liquid form of cement is achieved, then the agent should be added [41]. The optimal antibiotic-to-cement ratio is suggested to be 10–15% (weight/weight) to maintain antibiotic levels above the minimum inhibitory concentration at the spacer-body interface for six weeks [42,43]. Higher antibiotic concentrations can reduce the mechanical properties of the cement [44]. The highest recommended antibiotic-to-cement ratio is 8/40 (weight/weight) [45].

### 4.2. Dosage of Antibiotics in Cement Spacers

The dosage of the antibiotic varies depending on the intended use of the cement. Types of cement containing 2 g of antibiotic per 40 g of cement are categorised as low-dose antibiotic-loaded cement, whereas those with higher antibiotic concentrations are considered high-dose antibiotic-loaded cement [26,46]. Many researchers suggest that high doses of antibiotics should be used in cases of acute infections: more than 2 g per 40 g of cement, typically ranging from 6 to 8 g per 40 g, to ensure prolonged and effective release against pathogens [47,48,49]. Conversely, when the ALCS is employed for prophylaxis during initial implants, where the primary function of the cement is to secure the implant, antibiotics can be mixed at lower doses: less than 2 g per 40 g of antibiotic cement [47,48,49].

### 4.3. Types of Antibiotics in Cement Spacers

**Figure 1:**
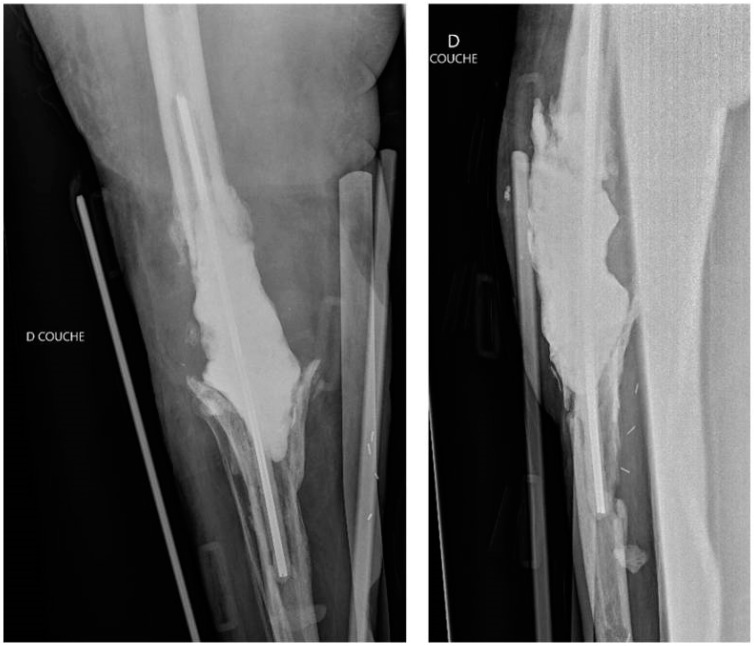
An example of a hand-made static antibiotic-loaded cemented spacer in a case of severe periprosthetic joint infection. Anteroposterior and lateral post-operative radiographic views of the right knee after removal of the infected implants and placement of a cemented spacer loaded with vancomycin and gentamycin, fixed with two metal rods [50]. [Authors’ previously published work, used under the terms and conditions of the Creative Commons Attribution (CC BY) license (https://creativecommons.org/licenses/by/4.0/, accessed on 10 August 2024).].

Spacers may contain multiple antibiotics [5,11]. When the causative bacteria are identified and sensitive to a specific antibiotic, using a single agent may be sufficient [11]. However, when the bacteria are unknown, it is preferable to combine antimicrobials to cover a broader spectrum, including both Gram-positive and Gram-negative bacteria [5,11]. The antibiotics most commonly used for cement impregnation are tobramycin, gentamicin, vancomycin, and cephalosporins [5]. Vancomycin is effective against MRSA, gentamicin targets *Enterobacteriaceae* and *Pseudomonas aeruginosa*, and cefotaxime is effective against gentamicin-resistant bacteria [5]. A popular combination is an aminoglycoside (tobramycin or gentamicin) with vancomycin [11,18,26] (Figure 1).

Using a combination of antibiotics broadens the antimicrobial spectrum and enhances the release of both antibiotics, a phenomenon known as passive opportunism [5,29]. Combining an aminoglycoside with vancomycin is recommended even if the bacteria are resistant to aminoglycosides, as high concentrations of aminoglycosides boost vancomycin release [11]. The combination of meropenem with vancomycin also increases vancomycin release [51]. The mechanical properties of commercially available bone cement vary depending on its brand and composition, with compressive strengths typically ranging between 80 and 100 MPa. Table 3 presents a list of commercially available types of bone cement that incorporate various antibiotics [34,52].

Apart from the commonly used antibiotics (vancomycin, tobramycin, and gentamicin), other suitable antimicrobial regimens for cement impregnation are presented in Table 4. Rifampicin, though highly effective against *S. aureus* biofilms, is not suitable for spacer impregnation as it compromises the cement’s mechanical properties [54]. Tetracycline is effective against both Gram-positive and Gram-negative bacteria, but it is not very heat-resistant and may promote bacterial resistance [5,18,29].

An in vitro study examined the effectiveness of three antibiotic-loaded cements (daptomycin, vancomycin, and teicoplanin) against different strains of *Staphylococcus aureus*, including methicillin-susceptible, methicillin-resistant, and vancomycin-intermediate strains [55]. While all three antimicrobial agents maintained their ability to kill bacteria, teicoplanin-loaded cement showed superior elution and a longer period of inhibition, whereas vancomycin-loaded cement exhibited a 21-day antibacterial effect [55]. Meropenem was found to elute in effective concentrations from cement, remaining active against MRSA, *Pseudomonas aeruginosa*, *Escherichia coli*, and *Klebsiella pneumonia* for a period ranging from 3 to 27 days [56]. Combining two antibiotics has been demonstrated to be more effective than using a single antibiotic, with the combination of meropenem and vancomycin showing broad-spectrum activity and enhancing vancomycin elution [51,57].

### 4.4. Antifungal-Loaded Bone Cement

Fungal PJIs are rare but severe, while their incidence has risen in recent decades due to an ageing population and an increased number of immunosuppressed individuals [50,58,59]. Despite the widespread clinical application of ALCS, the utility of antifungal-impregnated bone cement remains controversial [58,60]. The optimal characteristics of antifungal agents for incorporation into bone cement have yet to be defined [58,61,62]. Presumably, these characteristics should resemble those of antibiotics, including availability in powder form, a broad antifungal spectrum, fungicidal properties at low concentrations, sustained release from bone cement, thermal stability, minimal risk of allergy or delayed hypersensitivity, negligible impact on cement mechanical properties, and low serum protein binding [26].

The bulk of knowledge regarding antifungal-loaded bone cement stems from experimental investigations. Among the studied agents; amphotericin B, voriconazole, and fluconazole have received the most attention [63,64,65,66,67,68,69]. Silverberg et al. demonstrated the ongoing efficacy of fluconazole and amphotericin B post-cement polymerization, contrasting with fluorocytosine, which lost its activity [69]. Regarding amphotericin B utilisation, Goss et al. reported limited elution with a cumulative release rate below 0.03% of the incorporated amount [70]. Conversely, Houdek et al. observed sufficient deoxycholate amphotericin B release from beads in vitro [71]. Cunningham et al. noted enhanced release from bone cement with liposomal amphotericin B compared to deoxycholate amphotericin B, a finding corroborated by Czuban et al. [72,73]. Although this liposomal formulation of amphotericin B demonstrated safety in a mouse model, it exhibited cytotoxicity under in vitro conditions [74].

Limited experience exists primarily with fluconazole, voriconazole, and amphotericin B, making them potential candidates for impregnating bone cement. However, it is essential to recognise that conclusions drawn from in vitro studies may not directly translate to in vivo outcomes. Factors such as the type, quantity, and proportion of agents used, the cement’s type and porosity, surface characteristics, preparation method, and environmental conditions can all influence the elution kinetics of bone cement [75]. Consequently, there is no consensus on the optimal type and dosage of antifungal agents for clinical use against PJIs.

Future studies should explore the optimal impregnation of antifungal agents to ensure clinical efficacy and infection eradication. Inadequate impregnation of cement beads or spacers could negatively impact the treatment outcome and increase the risk of infection persistence, particularly if fungal organisms survive surgical debridement and develop resistance due to subinhibitory concentrations of locally released antifungals. Additionally, since biofilms form in fungal infections similar to bacterial infections, the risk of resistance development is a concern. Studies have shown that *Candida albicans* produces more biofilm than other *Candida* species and rapidly develops resistance to fluconazole [76].

## 5. Mechanical Properties of Antimicrobial Cement

PMMA cements must demonstrate adequate resistance against various forces including bending, impact, tension, torsion, and shear forces [53]. According to ISO standards, commercially available antibiotic-loaded bone cement (ALBCs) should have a bending modulus of at least 1800 MPa and a bending strength of 50 MPa [53]. The compression test determines the maximum compressive force a cylindrical specimen can withstand before undergoing plastic deformation. The mechanical properties of commercially available bone cement vary depending on its brand and composition, with compressive strengths typically ranging between 80 and 100 MPa.

The inclusion of antibiotics diminishes mechanical stability due to the interference of antibiotic molecules with the cement curing process. Liquid antibiotics, in particular, significantly disrupt the polymerization process. For instance, Hsieh et al. noted a 37% decrease in compressive strength when liquid gentamicin was incorporated into Simplex bone cement (Stryker, Kalamazoo, MI, USA) [29]. Consequently, it is recommended, as a general rule, to solely employ crystalline antibiotics and add them to the powder component [26,76]. The impact on mechanical properties not only hinges on the physical state of the antibiotic but also its specific type. Thus, it is advisable to utilise antibiotics that have been determined, through in vitro studies, to be suitable for manual loading.

## 6. The Role of Antimicrobial Cement in Two-Stage Exchange Arthroplasty and Double DAIR Procedure

Alongside antimicrobial therapy, surgical intervention is a key component in managing PJIs [77]. Surgical approaches include DAIR, as well as one- and two-stage exchange procedures. Selection of the optimal surgical technique depends on factors such as the presentation and duration of the infection, host and local considerations, and institutional or surgeon expertise [78,79,80].

The two-stage exchange involves removing the prosthesis during the first stage and inserting an antibiotic-loaded cement spacer. After four to six weeks of systemic antibiotic treatment, infection control is typically achieved. The two-stage exchange has demonstrated efficacy, with reported success rates ranging between 75% and 98% in eliminating infections [77].

Under certain circumstances, there arises a need for the interim exchange of the initial antibiotic spacer. For example, persistent infection, spacer displacement, or fracture may necessitate an additional surgical intervention known as “spacer exchange” [77]. In cases of persistent infection, the rationale for spacer exchange surgery involves interim irrigation, debridement, and the administration of an additional antimicrobial load through antibiotic-impregnated spacers. If spacer exchange becomes necessary, the likelihood of completing the second stage decreases significantly [77].

### 6.1. Impact of Spacer Exchange on the Success of Two-Stage Revision

To identify predictors of success in a two-stage revision, Mortazavi et al. [81] analysed prospectively collected data from a cohort of 117 hips and knees undergoing two-stage exchange. The authors found that PJIs without isolation of pathogen, infection from MRSA, and prolonged reimplantation operative time were predictive of failure. Although Mortazavi et al. did not observe any correlation between preoperative serum erythrocyte sedimentation rate (ESR) and C-reactive protein (CRP) levels at the time of reimplantation and failure of two-stage exchange, Dwyer et al. [82] reported that patients with a preoperative serum ESR >99 mm/h had a 1.8 times higher risk of failure. Additionally, Dwyer et al. found that patients with synovial fluid white blood cell (WBC) count >60,000 cells/μL and synovial fluid neutrophils >92% were at 2.5 and 2.0 times higher risk of failure, respectively. In 2013, Diaz-Ledezma et al. [83] delineated criteria for defining the successful treatment of PJI following a two-stage exchange. These criteria were composed of the following: (1) eradication of infections, evidenced by a healed wound without fistula, drainage, or pain, and absence of recurrent infection caused by the same organism strain; (2) absence of subsequent surgical interventions for infection following reimplantation surgery; and (3) no occurrences of PJI-related mortality (e.g., sepsis or necrotizing fasciitis). However, these criteria were formulated based on outcomes post-reimplantation, disregarding the interim period and patients who did not undergo reimplantation. The diverse outcomes observed throughout a two-stage exchange underscore the necessity of establishing new criteria for success/failure that do not solely rely on reimplantation as the starting point.

Hence, the International Consensus Meeting in Musculoskeletal Infection in 2018 advocated for the commencement of evaluation from the first stage, encompassing the removal of the infected implant and insertion of the spacer [52,84]. The consensus [52,84] also proposed categorizing outcomes of a two-stage exchange into three groups: success, failure due to secondary causes, and failure due to PJI. Success is defined as the resolution of PJI without subsequent interventions. Failure due to secondary causes encompasses aseptic or septic revisions (including DAIR, but excluding amputation, resection arthroplasty, and fusion) occurring after 1 year or mortality after 1 year. On the other hand, failure related to PJI, either directly or indirectly, is characterised by aseptic or septic revision within 1 year, salvage procedures (such as amputation, resection arthroplasty, or arthrodesis), retention of the spacer, or death within 1 year of initiating PJI treatment.

### 6.2. The Double-DAIR Procedure

DAIR procedures present varying success rates in treating acute periprosthetic joint infection (PJI), with generally unfavourable outcomes documented in the literature [85]. Due to DAIR’s notably high failure rate, a two-stage debridement approach incorporating high-dose antibiotic beads between stages for managing acute PJI was adopted [85,86,87]. During the double DAIR procedure, upon joint exposure, cultures are obtained, and all modular components are removed, cleaned, and soaked in an antiseptic solution. Thorough irrigation and debridement with complete synovectomy follow, with temporary reinsertion of the original modular parts. High-dose antibiotic cement beads are introduced into the joint before closure. Around 5 to 6 days later, a second debridement is conducted, the beads are removed, and new sterile modular components are implanted. The patient receives a course of intravenous antibiotics initially, followed by oral antibiotics, alongside standard postoperative rehabilitation [85,86,87].

The double-DAIR procedure has demonstrated consistent success, with infection-control rates of 87% and 90% reported in two studies from a single cohort at our institution [86,87]. These rates indicate a significant improvement compared to single irrigation and debridement methods and are comparable to those reported for two-stage exchange arthroplasty procedures [85]. Table 5 presents the advantages of using beads or spacers in the double DAIR procedure.

## 7. Complications and Toxicity

Complications related to ALCSs are quite common, affecting 26–58.5% of patients undergoing staged revision arthroplasty [5,10,12,88]. These complications can be categorised as either local or systemic. Articulating the ALCS may damage the knee extensor mechanism and result in wound dehiscence [5]. Additionally, remaining small abrasive ALCS particles may lead to failure of the final prosthesis [26]. Static ALCSs may contribute to bone loss [5], while hip ALCSs can cause periprosthetic femur fractures. Tilting of the spacer due to poor fit in the proximal femur may result in dislocation or subluxation of the ALCS [5,10,12,77].

Systemic complications following ALCS implantation are linked to the high release of antibiotics immediately after the first-stage surgery. These complications include nephrotoxicity, hepatotoxicity, and ototoxicity due to gentamicin, allergic reactions, and neutropenia due to vancomycin [18,26]. Older patients are believed to be more susceptible to such complications [26]. In some cases, ALCS removal becomes necessary to restore renal function [5,88,89]. Notably, a significant increase in serum creatinine, exceeding 50% of normal levels, has been reported in 20% of patients with tobramycin-impregnated spacers during the initial postoperative week following the first-stage surgery [90]. Underlying renal dysfunction predisposes individuals to ALCS-related nephrotoxicity [5,90]. However, some studies suggest that the elevated antibiotic levels observed immediately after spacer implantation return to normal within 24 h, mitigating the risk of systemic side effects [5,29].

Antibiotics incorporated into cement can potentially harm cells involved in tissue regeneration, such as osteoblasts, endothelial cells, fibroblasts, or skeletal muscle cells [91]. Elevated antibiotic concentrations can be particularly detrimental to tissue cells, including osteoblasts; thereby, affecting bone formation, especially in the context of the ALCS due to compromised vascularization [34,92]. Different antimicrobial agents exhibit varying levels of toxicity. For instance, vancomycin, tobramycin, and gentamicin, commonly used in bone cement, have distinct toxicity profiles towards osteoblasts. At concentrations exceeding 2000 µg/mL, vancomycin and tobramycin show lower toxicity towards osteoblasts compared to gentamicin [92]. Vancomycin affects cell death and osteogenic activity at doses of 5000 µg/mL, while tobramycin impacts cell replication at concentrations above 500 µg/mL, with significant cell death observed at 5000 µg/mL [92].

**Figure 2:**
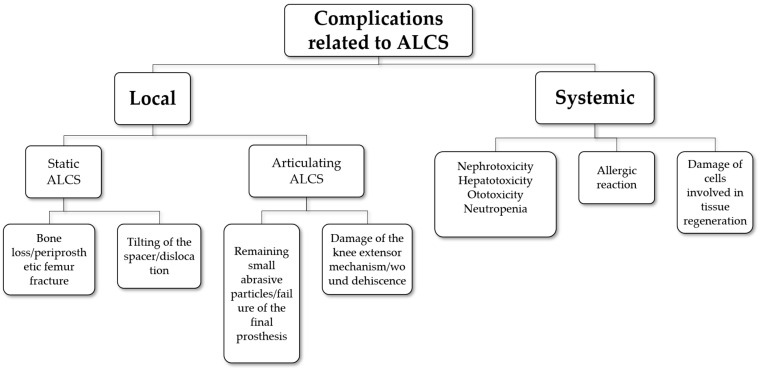
Complications related to antibiotic-loaded cement spacers (ALCSs).

The toxicity profiles of antibiotics can vary depending on the cell types and experimental methodologies, including exposure duration. Another critical factor influencing antibiotic toxicity towards tissue cells is the duration of exposure and its intracellular accumulation, particularly in the case of high-dose bone cement. For instance, repeated clindamycin and erythromycin exposure on osteoblasts have demonstrated intracellular antibiotic accumulation, resulting in much lower concentrations compared to single applications [34]. Figure 2 summarises the ALCS-related complications.

The surface of bone cement promotes microbial adherence and colonization, even in the presence of antibiotics [5]. The risk of microbial resistance may escalate when the antibiotic concentration falls below therapeutic thresholds [5,18]. This issue has two dimensions: Concerning cemented primary total joint replacement, it remains uncertain whether implant fixation with the ALCS heightens the risk of microbial resistance, potentially predisposing to future PJIs with resistant microbes. While some authors argue that prophylactic gentamicin cement impregnation raises the likelihood of subsequent PJIs with gentamicin-resistant microbes, others disagree. Prophylactic ALCSs during the primary procedure do not appear to alter the microbe pattern in future PJIs [5,93]. Regarding two-stage revision arthroplasty for PJI, there is debate over whether ALCSs could foster bacterial resistance and secondary superinfection. Ma et al. demonstrated the presence of bacterial 16s rRNA on spacers retrieved during second-stage PJI surgery, suggesting viable bacteria were present on the ALCS during the “drug holiday” preceding second-stage surgery. These bacteria, termed “bacterial resisters”, were shielded within well-formed biofilms and could serve as a source of recurrent infection, potentially contributing to second-stage implantation failure [5,94]. However, there is no clinical evidence to suggest that using ALCSs in prophylaxis or treatment of PJIs and chronic osteomyelitis leads to increased antibiotic resistance.

## 8. Future Perspectives

PJIs continue to pose significant challenges in orthopaedic surgery and, hence, it is essential to enhance the effectiveness of ALCSs [5]. Ongoing research initiatives are focused on surface modification and microencapsulation of specific antibiotics, such as rifampicin, to facilitate biofilm breakdown [95]. Moreover, apart from advancing antibiotic-loaded PMMA for preventing and treating PJIs, exploring new carriers and coatings for controlled release of local antimicrobial agents has also gained popularity in the last few years. These novel carriers also hold promise for prophylactic applications with uncemented prostheses in total joint arthroplasty [96]. Examples of novel surface modifications include antibiotic-releasing biodegradable polymers, antibiotic-releasing hydroxyapatite, vancomycin-loaded chitosan, and antibiotic-loaded hydrogels [18,96].

Taking into account the escalating issue of antibiotic-resistant bacteria, it is of utmost importance to explore non-antibiotic preventive and treatment strategies to forestall biofilm formation leading to subsequent PJIs or chronic osteomyelitis. In PJI prevention, coated implants with silver nanoparticles or silver ions, employing the inorganic antimicrobial Novaron, or harnessing antimicrobial peptides represent current developments that have shown promising outcomes in preclinical stages [97,98,99].

## 9. Conclusions

Various types of spacers are available, including pre-manufactured or handmade, static or articulating. These spacers help maintain limb anatomy and function until the final prosthesis can be inserted. Most importantly, ALCSs are effective carriers of antimicrobial agents, delivering local antibiotic concentrations far exceeding those achievable through systemic administration, without the risk of systemic toxicity. Surgeons must consider all factors affecting antibiotic release from ALCS, as well as the mechanical properties of antimicrobial cement, to make an appropriate choice. Spacers release high levels of antibiotics directly in contact with infected bone and soft tissues to eradicate biofilm-protected microbes. Selecting the correct type and amount of antibiotics in the spacer is vital for infection control. Potential complications of spacers, both local and systemic, must also be considered. Patients with comorbidities, resistant bacteria, or multiple previous surgeries at the joint are at higher risk for failure of final prosthesis insertion and may require salvage surgery. As PJIs remain significant challenges in orthopaedic surgery, it is essential to improve treatment methods using ALCS. Additionally, due to the growing issue of antibiotic-resistant bacteria, there is a need to explore non-antibiotic preventive and treatment strategies to prevent biofilm formation and subsequent PJIs or chronic osteomyelitis.

## Figures and Tables

**Table 1:** Types of Articulating Spacers.

Types of Articulating Spacers	Characteristics	
	Advantages	Disadvantages
Hand-made spacers [24]	Lower cost, custom-made to suit specific anatomical configurations, can be loaded with antibiotics that are more sensitive to pathogens	Time-consuming, heightened risk of fracture, potential mismatch with articulating surfaces
Spacers fashioned from moulds [24]	More consistent geometry, and various sizes, allowing intraoperative selection, can be loaded with antibiotics that are more sensitive to pathogens	Increased procedure costs, higher incidence of mechanical complications
Commercial preformed spacers [24]	Straightforward intraoperative application, time-saving	Lower antibiotic doses and limitations on the types of antibiotics used
Spacers with additional metal or polyethylene articulating elements [24]	Semi-constrained to minimise dislocation risk, advocated for complex cases	Limited availability
New or autoclaved prosthesis [24]	Significant flexibility in two-stage exchange procedures, allowing partial prosthesis removal or combination with other strategies to manage complications	It contradicts recommendations from regulatory bodies like the Food and Drug Administration (FDA) in the USA and the Medicines and Healthcare Products Regulatory Agency (MHRA) in the UK
Custom-made articulating spacers (CUMARS) [24]	Better joint function, enabling full weight-bearing of the lower extremity, improved inter-stage functionality, easier removal, and effective infection control	High cost
3D printing-assisted articulating spacers [24]	High geometric consistency, different-sized models while preserving geometry, minimal mechanical complications	Lack of relevant medical regulations and consensus

**Table 2:** Factors affecting antibiotic release from ALCS.

Increasing Antibiotic Release	Decreasing Antibiotic Release
Greater the surface area [5]	Increased distance between the cement and surrounding tissues
Cancellous bone/dead space	Cortical bone
Porosity/ Intact crystals left from the mixing process	
Partially mixed cements	
Joint motion	
Tissue absorption capacity	
Increased concentration of the antibiotic	

**Table 3:** Commercially available types of bone cement incorporated in various antibiotics, adapted from ICM [34,53]. Used under the terms and conditions of the Creative Commons Attribution (CC BY) license (https://creativecommons.org/licenses/by/4.0/) accessed on 10 August 2024.

Cement	Antimicrobial Agents	Coverage Spectrum
Palacos	Gentamicin	Gram-negative (*Pseudomonas*, *Proteus*, *Klebsiella*, *E. coli*), Gram-positive *Staphylococcus*, aerobic bacteria
CMW/Simplex/Palacos	Gentamicin	Gram-negative (*Pseudomonas*, *Proteus*, *Klebsiella*, *E. coli*), Gram-positive *Staphylococcus*, aerobic bacteria
Palacos	Gentamicin/erythromycin/penicillin	Gram-negative (*Pseudomonas*, *Proteus*, *Klebsiella*, *E. coli*, *Mycoplasma*, *Chlamydia trachomatis*, *Rickettsia*), Gram-positive cocci, aerobic bacteria, aerobic bacilli
Simplex	Penicillin/methicillin/erythromycin/lincomycin/nafcillin/	Gram-negative (*Pseudomonas*, *Proteus*, *Klebsiella*, *E. coli*, *Mycoplasma*, *Chlamydia trachomatis*, *Rickettsia*, *Treponema*), Gram-positive cocci, aerobic bacteria, aerobic bacilli
	polymyxin/colistimethate	Gram-negative (jnc. *Pseudomonas*)
Simplex/Palacos	Gentamicin/oxacillin/cephazolin	Gram-negative (*Pseudomonas*, *Proteus*, *Klebsiella*, *E. coli*), Gram-positive cocci, aerobic bacteria
Palacos/Simplex/CMW	Sodium fusidate/gentamicin	Gram-negative (*Pseudomonas*, *Proteus*, *Klebsiella*, *E. coli*), Gram-positive cocci, aerobic bacteria
Simplex	Fucidin/clindamycin/gentamicin	Gram-negative (*Pseudomonas*, *Proteus*, *Klebsiella*, *E. coli*, *Mycoplasma*, *Chlamydia trachomatis*, *Rickettsia*), Gram-positive cocci, aerobic and anaerobic bacteria and bacilli
Palacos/Simplex	Penicillin/gentamicin	Gram-negative (*Pseudomonas*, *Proteus*, *Klebsiella*, *E. coli*, *Mycoplasma*, *Chlamydia trachomatis*, *Rickettsia*, *Treponema*), Gram-positive cocci, aerobic bacteria, aerobic bacilli
Palacos/CMW	Gentamicin sulphate/sodium fusidate/diethanolamine	Gram-negative (*Pseudomonas*, *Proteus*, *Klebsiella*, *E. coli*)
Palacos/CMW	Ceftriaxone/coumermycin/sulfampicionmethoxozaole/	Gram-positive bacteria, Gram-negative bacteria (*Klebsiella*, *E. coli*, *Proteus*, not including *Pseudomonas)*
	Trimethoprim/cephalothin/vancomycin/fusidic acid/	Gram-positive bacteria (including methicillin-resistant organisms), Gram-negative bacteria (esp. *Pseudomonas*)
	Gentamicin/rifampicin/vancomycin	Gram-positive bacteria (including methicillin-resistant organisms), Gram-negative bacteria (esp. *Pseudomonas*), *Mycobacterium*
Palacos/Simplex/Zimmer	Vancomycin/amikacin/daptomycin	Gram-positive bacteria (including methicillin-resistant organisms), Gram-negative bacteria (esp. *Pseudomonas*)
low viscosity and dough type		
Palacos/Simplex	Tobramycin/vancomycin	Gram-positive bacteria, (including methicillin-resistant organisms), Gram-negative bacteria (especially *Pseudomonas*)
Palacos	Vancomycin/tobramycin	Gram-positive bacteria, (including methicillin-resistant organisms), Gram-negative bacteria (especially *Pseudomonas*)
Cerafix	Vancomycin	Gram-positive bacteria (including methicillin-resistant organisms)

**Table 4:** List of antimicrobial agents suitable for use as spacers [53]. Used under the terms and conditions of the Creative Commons Attribution (CC BY) license (https://creativecommons.org/licenses/by/4.0/) accessed on 10 August 2024.

Antibiotics	Dose per 40 g Cement
Tobramycin	1 to 4.8 g
Gentamicin	0.25 to 4.8 g
Cefazolin	1 to 2 g
Cefuroxime	1.5 to 2 g
Ceftazidime	2 g
Cefotaxime	2 g
Ceftaroline	2 to 4 g
Ciprofloxacin	0.2 to 3 g
Vancomycin	0.5 to 4 g
Clindamycin	1 to 2 g
Erythromycin	0.5 to 1 g
Colistin	0.24 g
Piperacillin—not available Piperacillin-tazobactam	4 to 8 g
Aztreonam	4 g
Tazobactam	0.5 g
Linezolid	1.2 g
Meropenem	0.5 to 4 g
Daptomycin	2 g
Amphotericin	200 mg
Voriconazole	300–600 mg
Itraconazole	250 mg
Fluconazole	200 mg

**Table 5:** Pros and cons of antibiotic-loaded PMMA beads and spacers [18] (Used under the terms and conditions of the Creative Commons Attribution (CC BY) license (https://creativecommons.org/licenses/by/4.0/) accessed on 10 August 2024.

Beads or Bead Chains		Spacers	
Pros	Cons	Pros	Cons
Increased antibiotic concentrations	Disruption of local anatomy	Preservation of local anatomy	Lower antibiotic concentrations and shorter duration
Cost-effective	Extensive scar tissue formation	Pre-fabricated spacers are costly	Spacer dislocation/ migration
Low complication rates		Possible joint mobility and weight-bearing	Spacer fractures
Simple to use, implant, and remove			
Longer period at minimum inhibitory concentration (MIC)			

## Data Availability

The data presented in this study are available in the article.
